# Supplementary material for: Surface Hygiene Evaluation Method in Food Trucks as an Important Factor in the Assessment of Microbiological Risks in Mobile Gastronomy
Source: Foods. 2023 Feb 10;12(4):772. doi: 10.3390/foods12040772 (PMC9955632; doi:10.3390/foods12040772)
Supplement: Supplementary file 1 [file foods-12-00772-s001.zip › foods-2192657-supplementary.pdf]

Table S1. Total Viable Count result on five different surfaces in analysed food truck outlets

FT	Refrigerator*		Cutting board*		Knife*		Serving board*		Working board*	
	Mean P<br>SD	Mean R<br>SD	Mean P<br>SD	Mean R<br>SD	Mean P<br>SD	Mean R<br>SD	Mean P<br>SD	Mean R<br>SD	Mean P<br>SD	Mean R<br>SD
FT1	2.06$^{4,6-16,19,20}$<br>±0.11	1.69^{13,14,19}<br>±0.27	1.96<br>±0.41	0.73^{5,6,10}<br>±0.36	1.94$^{3-20}$<br>±0.23	2.17^{9,10,13,14,19}<br>±0.37	2.68^{2}<br>±0.45	1.45$^{3,4,8-10,14,16}$<br>±0.21	2.62^{15}<br>±0.51	0.72$^{2-5,7,9,10,15,15,18}$<br>±0.17
FT2	1.39$^{4,7-12,15,16}$<br>±0.13	1.20^{17}<br>±0.50	4.74<br>±1.77	1.25$^{3,7-9,14,15,17}$<br>±0.23	2.24$^{3-6,8-11,13-15,17-19}$<br>±0.13	1.60$^{6,8-14,19}$<br>0.56	2.49$^{1-20}$<br>±0.08	5.22$^{3,4,8,10,14-16}$<br>±0.95	5.57^{15}<br>±0.95	1.25$^{1,6-9,11-20}$<br>±0.18
FT3	1.01$^{4,6-13,15-17}$<br>±0.15	1.50$^{10, 16-18, 20}$<br>±0.03	0.95<br>±0.06	1.24^{2,5,6,10}<br>±1.08	1.2^{1,2,10}<br>±0.26	0.60^{10,13,19}<br>0.43	6.63^{2,5,18}<br>±0.66	0.15$^{1,2,5-9,11-20}$<br>±0.21	5.27^{15}<br>±0.40	0.92$^{1,6-9,11-20}$<br>±0.11
FT4	1.78$^{1-5, 17-18}$<br>±0.14	nd<br>-	2.65$^{6-10, 12-18}$<br>±0.35	2.66^{5,6,10}<br>±0.02	0.89^{1,2,10,12,20}<br>±0.28	nd<br>-	5.39^{2,5}<br>±1.17	0.44$^{1,2,5-9,11-20}$<br>±0.23	6.72$^{15-16}$<br>±0.55	0.48$^{1,6-9,11-20}$<br>±0.51
FT5	1.83$^{4-16,19,20}$<br>±0.09	1.78^{14,19}<br>±0.03	6.19^{4}<br>±1.39	1.49$^{1,3,4,7-9,11-20}$<br>±0.01	1.41^{1,2,10}<br>±0.15	0.15^{10}<br>±0.21	2.26$^{2-7,9,11,13,15,17,20}$<br>±0.26	2.14^{3,4,8,10,16}<br>±0.11	6.50^{15}<br>±0.40	0.60$^{1,6-9,11-20}$<br>±0.42
FT6	1.57^{1,3,5,17,18}<br>±0.14	0.39$^{14, 17, 19}$<br>±0.13	5.94^{4}<br>±1.48	0.30$^{1,3,4,7-9,11-20}$<br>±0.42	0.57^{1,2,10,12,20}<br>±0.39	nd<br>-	1.71^{2,5}<br>±0.10	0.48^{3,4,8}<br>±0.67	1.23^{15}<br>±0.16	0.74$^{2-5,10,19}$<br>±0.37
FT7	1.22$^{1-3,5,18,19}$<br>±0.30	nd<br>-	0.88^{4}<br>±0.15	nd<br>-	1.61^{1,10}<br>±0.54	0.68^{10,13,19}<br>±0.10	1.86^{2,5}<br>±0.21	0.39^{3,4,8}<br>±0.12	0.65^{15}<br>±0.28	0.63$^{1-5,10,16-17,19,20}$<br>±0.46
FT8	0.92$^{1-3,5,18,19}$<br>±0.10	nd<br>-	0.74^{4}<br>±0.20	0.54^{2,5,6,10}<br>±0.34	0.58^{1,2,10}<br>±0.82	0.15^{2,10,16,20}<br>±0.21	0.40^{2}<br>±0.57	1.17$^{1-5,10,17-20}$<br>±0.18	1.65$^{15-16}$<br>±0.14	0.45$^{1-5,10}$<br>±0.20
FT9	1.08$^{1-3,5,18,19}$<br>±0.18	nd<br>-	nd<br>-	0.30^{2,5,6,10}<br>±0.42	0.38^{1,2,10,12,20}<br>±0.14	0.00<br>-	0.83^{2,5}<br>±0.10	0.57^{1,3,4,10,20}<br>±0.14	0.38$^{15-16}$<br>±0.54	0.44$^{1-5,10,13,16-17,19-20}$<br>±0.62
FT10	4.42$^{1-3,5,18,19}$<br>±0.68	nd<br>-	5.80^{4}<br>±1.27	0.00<br>±0.00	6.25$^{1-20}$<br>±0.92	5.87$^{1-20}$<br>±0.44	6.73^{2,5,18}<br>±0.40	0.00<br>-	6.93$^{15-16}$<br>±0.38	0.50$^{1,6-20}$<br>±0.28
FT11	2.06$^{1-3,5,18,19}$<br>±0.24	nd<br>-	2.54^{4}<br>±0.50	1.32^{5,6,10}<br>±0.06	0.75^{1,2,10}<br>±0.07	0.50^{2,10,16,20}<br>±0.28	1.73^{2,5}<br>±0.23	0.39^{3,4,10}<br>±0.12	1.21$^{15-16}$<br>±0.13	0.50$^{2-5,10,19}$<br>±0.70
FT12	1.67$^{1-3,5,18,19}$<br>±0.31	nd<br>-	2.02^{4}<br>±0.73	nd<br>-	0.74$^{1,4, 6,9, 10,13-15,17-19}$<br>±0.20	0.94^{2,10,16,20}<br>±0.14	1.68^{2}<br>±0.27	0.86^{3,4,10}<br>±0.12	2.05^{15}<br>±0.07	0.78$^{2-5,10,18}$<br>±0.43
FT13	0.88^{1,3,5,18,19}<br>±0.14	0.63$^{1, 17-18,20}$<br>±0.21	1.76^{4}<br>±0.19	0.64^{5,6,10}<br>±0.91	nd<br>-	nd<br>-	1.98^{2,5}<br>±0.23	0.35^{3,4,10}<br>±0.49	2.27^{15}<br>±0.24	1.13$^{2-5,9,10,14,18}$<br>±0.61
FT14	0.33^{1,5,18,19}<br>±0.47	0.70$^{1,5,6, 17,18,20}$<br>±0.00	0.45^{4}<br>±0.64	nd<br>-	0.35^{1,2,10,12,20}<br>±0.49	nd<br>-	0.64^{2,5}<br>±0.48	0.30$^{1-4,10,17-20}$<br>±0.42	0.45$^{15-16}$<br>±0.64	0.50$^{1-5,10,13,16-17,19-20}$<br>±0.71
FT15	1.67$^{1-3,5,18,19}$<br>±0.33	nd<br>-	1.69^{4}<br>±0.43	nd<br>-	1.62^{1,2,10,12,20}<br>±0.22	nd<br>-	0.66^{2,5}<br>±0.17	0.45$^{1-4,10,17,19,20}$<br>±0.64	0.92$^{1-20}$<br>±0.22	6.57$^{1-5,10,16,19-20}$<br>±0.63
FT16	2.14$^{1-3,5,18,19}$<br>±0.28	nd<br>-	2.82^{4}<br>±0.30	0.56^{5,6,10}<br>±0.79	2.39^{1,10}<br>±0.30	0.72$^{4,6,8-14,17,19}$<br>±0.17	0.44^{2}<br>±0.62	0.69$^{1-4,10,17-20}$<br>±0.13	2.88$^{4,8-11,14,15,19}$<br>±0.32	2.26$^{2-5,7,9,10,14,15,18}$<br>±0.25
FT17	2.92$^{2-5,6-16,19,20}$<br>±0.09	2.40^{2,6,13,14,19}<br>±0.57	1.01^{4}<br>±0.16	0.23^{2,5,6,10}<br>±0,32	0.79^{1,2,10,12,20}<br>±0.16	nd<br>-	2.56^{2,5}<br>±0.46	0,40$^{3,4,8,10,14-16}$<br>±0.57	2.52^{15}<br>±0.36	0.70$^{2-5,7,9,10,14,18}$<br>±0.56
FT18	2.28$^{4,6-16,19,20}$<br>±0.40	1.90$^{3, 13,14,19}$<br>±0.14	2.61^{4}<br>±0.23	0.33^{5,6,10}<br>±0.47	1.87^{1,2,10,12,20}<br>±0.10	nd<br>-	2.45^{2,5,10}<br>±0.44	1.74^{3,4,8,10,14,16}<br>±0.38	0.28^{15}<br>±0.39	0.89$^{1-5,10,12,13,16-20}$<br>±0.16
FT19	0.38^{1,5,17,18}<br>±0.11	0.81$^{1,4-6,17,18,20}$<br>±0.30	2.40<br>±0.07	1.34^{5,6,10}<br>±0.58	nd<br>-	nd<br>-	2.53^{2}<br>±0.21	0.65$^{3,4,8,10,14-16}$<br>±0.06	3.32^{15,16}<br>±0.19	0.43$^{2-7,9-11,14-15,18}$<br>±0.18
FT20	2.20^{1,5,17,18}<br>±0.18	0.70^{3,13,14,19}<br>±0.14	2.53<br>±0.21	1.60^{5,6,10}<br>±0.99	2.56$^{1,4, 6,9, 10,13-15,17-19}$<br>±0.08	0.99$^{4,6,8-14,17,19}$<br>±0.13	2.71^{2,5}<br>±0.26	0.20$^{3,4,8-10,14-16}$<br>±0.28	2.65^{15}<br>±0.35	1.19$^{2-5,7,9-10,14-15,18}$<br>±0.07

statistical differences in column (P<0.05); nd- not detected; P-Petrifim, R- reference method; FT – food truck

Table S2. *Staphylococcus aureus* result on five different surfaces in analysed food truck outlets

FT	Refrigerator*		Cutting board*		Knife		Serving board*		Working board*	
	Mean P SD	Mean R SD	Mean P SD	Mean R SD	Mean P* SD	Mean R SD	Mean P SD	Mean R SD	Mean P SD	Mean R SD
FT1	1.06 ±0.08	nd -	nd -	0.75^{10} ±0.64	nd -	0.15 ±0.21	nd -	$0.45^{3,8,12}$ ±0.64	$1.27^{3-5,7-12,14}$ ±0.05	1.06 ±0.08
FT2	Nd -	$0.74^{4,8,12}$ ±0.62	$1.67^{1,4,7,10,13,14}$ ±0.10	1.15 ±0.04	$1.06^{1,4,6-9,13}$ ±0.08	1.54 ±1.32	$1.06^{1,5,7,8,10-12,14}$ ±0.08	$0.80^{3,8,12}$ ±0.28	$1.55^{3-11,14}$ ±0.04	0.54 ±0.34
FT3	0.50^{6} ±0.71	$0.24^{4,8,12}$ ±0.34	$1.10^{1,7-9,12-14}$ ±0.14	$0{,}15^{9,10}$ ±0.21	0.66 ±0.26	1.31 ±1.85	$0.66^{1,5,7,8,11,12,14}$ ±0.26	$2.37^{1-7,9-11,13-15}$ ±0.17	nd -	0.63 ±0.21
FT4	0.74^{6} ±0.37	$1.98^{1-5,7,9-11,13-15}$ ±0.04	$0.81^{1,2,6-9,12-14}$ ±0.47	$0.48^{9,10}$ ±0.67	nd -	nd -	$0.81^{1,5,7,8,11,12,14}$ ±0.05	$0.24^{3,8,12}$ ±0.34	nd -	0.45 ±0.21
FT5	1.07 ±0.10	$0.89^{4,8,12}$ ±0.27	$1.50^{1,7-9,13,14}$ ±0.23	nd -	0.69 ±0.12	0.45 ±0.21	nd -	$0.65^{3,8,12}$ ±0.07	nd -	1.57^{13} ±0.55
FT6	$2.,11^{2-4,7-10,12-15}$ ±0.15	$1.06^{1,7,8,11,12,13-15}$ ±0.03	$1.59^{1,4,7-9,13-14}$ ±0.10	$0.39^{9,10}$ ±0.12	nd -	0.66 ±0.26	$1.10^{1,5,7,8,10-12,14}$ ±0.14	$0.15^{3,8,12}$ ±0.21	$0.80^{2-9,12,14}$ ±0.14	0.63 ±0.21
FT7	nd -	nd -	nd -	$0.60^{9,10}$ ±0.43	nd -	0.45 ±0.21	nd -	$0.54^{3,8,12}$ ±0.09	nd -	0.30 ±0.43
FT8	nd -	$2.49^{1-3,5-11,13-15}$ ±0.39	nd -	$0.39^{9,10}$ ±0.12	nd -	0.35 ±0.49	nd -	$2.57^{1,2,4-11,13-15}$ ±0.15	nd -	0.45 ±0.21
FT9	nd -	$0.50^{4,8,12}$ ±0.28	nd -	$1.95^{3-8,11-15}$ ±0.08	nd -	0.24 ±0.34	$1.09^{1,5,7-12,14}$ ±0.12	$0.50^{3,8,12}$ ±0.28	nd -	0.45 ±0.21
FT10	0.98^{6} ±0.03	$0.15^{4,8,12}$ ±0.21	$0.78^{1,2,7-9,12-14}$ ±0.43	$1.96^{1,3-8,11-15}$ ±0.20	$0.95^{1,4,6-10,13}$ ±0.07	0.30 ±0.43	$0.45^{2,6,9}$ ±0.21	$0.15^{3,8,12}$ ±0.21	$0.59^{1,2,12}$ ±0.16	0.50 ±0.28
FT11	$1.56^{2,7-9,12,14-15}$ ±0.17	nd -	$1.32^{1,7-9,13-14}$ ±0.20	$0.24^{9,10}$ ±0.34	0.65 ±0.49	0.50 ±0,28	nd -	$0.24^{3,8,12}$ ±0.34	$0.39^{1,2,12,13,15}$ ±0.55	0.35 ±0.49
FT12	nd -	$2.40^{1-3,5-7,9-15}$ ±0.18	2.04 ±0.07	$0.15^{9,10}$ ±0.21	$0.80^{1,4,6-10,13}$ ±0.28	0.77 ±0.10	nd -	$2.17^{1,2,4-7,9-15}$ ±0.08	$2.13^{1,3-15}$ ±0.03	0.24 ±0.34
FT13	0.74^{6} ±0.37	$0.15^{4,8,12}$ ±0.21	nd -	nd -	nd -	0.65 ±0.07	$0.80^{1,5,7,8,11,12,14}$ ±0.14	$0.15^{3,8,12}$ ±0.21	$1.07^{3-5,7-9,11-13}$ ±0.16	0.15^{5} ±0.21
FT14	nd -	nd -	nd -	$0.39^{9,10}$ ±0.12	0.30^{15} ±0.43	0.30 ±0.43	nd -	$0.15^{3,8,12}$ ±0.21	0.00 -	0.39 ±0.55
FT15	$0.35^{6,11}$ ±0.49	nd -	$1.09^{1,7-9,12-14}$ ±0.07	nd -	$1.42^{1,4,6-9,13,14}$ ±0.03	0.35 ±0.49	$0.63^{1,5,7,8,11,12,14}$ ±0.21	nd -	$1.03^{3-5,7-9,11,12,14}$ ±0.11	0,30 ±0.43

* statistical differences in column (P<0.05); nd- not detected; P-Petrifim, R- reference method; FT – food truck
